# Early evaluation of a natural language processing tool to improve access to educational resources for surgical patients

**DOI:** 10.1007/s00586-024-08315-5

**Published:** 2024-05-30

**Authors:** James Booker, Jack Penn, Kawsar Noor, Richard J. B. Dobson, Jonathan P. Funnell, Chan Hee Koh, Danyal Z. Khan, Nicola Newall, David Rowland, Siddharth Sinha, Simon C. Williams, Parag Sayal, Hani J. Marcus

**Affiliations:** 1 Wellcome/EPSRC Centre for Interventional and Surgical Sciences, University College London, London, UK; 2 Victor Horsely Department of Neurosurgery, National Hospital for Neurology and Neurosurgery, London, UK; 3 Institute for Health Informatics, University College London, London, UK; 4 NIHR Biomedical Research Centre, University College London Hospitals NHS Foundation Trust, London, UK; 5 Health Data Research UK London, University College London, London, UK; 6 NIHR Biomedical Research Centre, South London and Maudsley NHS Foundation Trust and King’s College London, London, UK; 7 Department of Biostatistics and Health Informatics, Institute of Psychiatry, Psychology and Neuroscience (IoPPN), King’s College London, London, UK; 8 Department of Neurosurgery, King’s College Hospital NHS Foundation Trust, London, UK; 9 Neurosciences Institute, Cleveland Clinic London, London, UK; 10 Department of Neurosurgery, The Royal London Hospital, London, UK

**Keywords:** Natural language processing, Education, Spine, Machine learning, Automation

## Abstract

**Purpose:**

Accessible patient information sources are vital in educating patients about the benefits and risks of spinal surgery, which is crucial for obtaining informed consent. We aim to assess the effectiveness of a natural language processing (NLP) pipeline in recognizing surgical procedures from clinic letters and linking this with educational resources.

**Methods:**

Retrospective examination of letters from patients seeking surgery for degenerative spinal disease at a single neurosurgical center. We utilized MedCAT, a named entity recognition and linking NLP, integrated into the electronic health record (EHR), which extracts concepts and links them to systematized nomenclature of medicine-clinical terms (SNOMED-CT). Investigators reviewed clinic letters, identifying words or phrases that described or identified operations and recording the SNOMED-CT terms as ground truth. This was compared to SNOMED-CT terms identified by the model, untrained on our dataset. A pipeline linking clinic letters to patient-specific educational resources was established, and precision, recall, and F1 scores were calculated.

**Results:**

Across 199 letters the model identified 582 surgical procedures, and the overall pipeline after adding rules a total of 784 procedures (precision = 0.94, recall = 0.86, F1 = 0.91). Across 187 letters with identified SNOMED-CT terms the integrated pipeline linking education resources directly to the EHR was successful in 157 (78%) patients (precision = 0.99, recall = 0.87, F1 = 0.92).

**Conclusions:**

NLP accurately identifies surgical procedures in pre-operative clinic letters within an untrained subspecialty. Performance varies among letter authors and depends on the language used by clinicians. The identified procedures can be linked to patient education resources, potentially improving patients’ understanding of surgical procedures.

**Supplementary Information:**

The online version contains supplementary material available at 10.1007/s00586-024-08315-5.

## Introduction

Informed consent is the foundation of shared decision making in surgery. It is defined by the patient being aware of any material risks involved in a proposed treatment [1, 2]. Despite this, patient recall after spinal surgery consent is only 45% immediately after discussion and lack of informed consent is a commonly cited reason for medicolegal claims [3–5]. This is particularly challenging in spinal surgery, which carries a wide range of risks, and the perceived benefits may not match patient expectations [6]. It is well-recognized that providing additional information through videos and online resources when consenting patients for neurosurgery improves understanding of the procedure and its risks [7, 8]. However, the heterogeneity of spinal surgery means that it is difficult for clinicians to provide additional information that is both informative and relevant to the patient’s specific situation.

Electronic health records (EHR) have quickly become established in modern healthcare settings as a safe, practical, and time efficient alternative to paper records [9, 10]. The use of EHR generates a wealth of easily accessible data, albeit in an unstructured form which makes it highly time and resource intensive to analyze. Natural language processing (NLP) is a subfield of artificial intelligence, which interprets and contextualizes written language [11]. The clinic letter remains the cornerstone of physician-to-physician communication, and as such contains a high density of patient specific information. However, the free text nature of clinic letters means that the information may be underexploited by standard digital automation, which analyses formal labels and ticked boxes (structured data), rather than unstructured data such as natural language. It remains unclear if NLP can be used in the retrieval of unstructured data from clinic spine surgery letters to build a diagnostic and surgical treatment profile for an individual, which can then be utilized to provide automated patient specific and personalized educational resources. An effective and scalable NLP could unburden clinicians from compiling, synthesizing, and recommending educational resources themselves [11]. These duties may shift clinicians’ focus away from the patient and contribute to a high clerical workload which can lead to burnout [12]. The NLP is designed for clinicians as a clinical tool to enhance the consent process. This could result in a more personalized consent process, empowering patients with individualized educational resources for better comprehension of their diagnosis and proposed treatment. This, in turn, may facilitate more focused and higher-level discussions during the formal consent consultation.

The study objectives are to evaluate the utility of an NLP model in identifying procedures from outpatient clinic letters and using a simple pipeline link these procedures to patient information using degenerative spinal disease as an exemplar.

## Methods

### Study design and methods summary

This study was a retrospective analysis of clinical records of patients before an elective spinal operation at a single neurosurgical center in the United Kingdom.

This study utilized the Medical Concept Annotation Toolkit (MedCAT)—an NLP tool within the data retrieval software CogStack—to recognize descriptions of surgical procedures for degenerative spine disease and to identify the Systematized Nomenclature of Medicine**—**Clinical Terms (SNOMED-CT) terms. To test the performance of MedCAT, one preoperative clinic letter per patient was manually labelled for surgical procedures by identifying and recording procedures described in the written text. MedCAT was used to link extracted procedure concepts to SNOMED-CT terms, which was compared against the labelled ‘ground truth’.

A pipeline was established to link clinic letters to relevant educational resources through a patient-specific dashboard integrated into the EHR.

For each task above (SNOMED identification and patient resource retrieval), macro-averaged precision, recall, and F1 scores were calculated for this linkage, using the actual procedure received by the patient as the ground truth.

The methods are described in more detail below.

### Participants

Patients were identified using a EHR system (Epic Caboodle, Epic Systems Corporation, Wisconsin, USA). The study population includes adult patients aged > 18 years old undergoing inpatient elective surgery for degenerative spine disease (both instrumented and non-instrumented), between January 2022 and June 2022. This study period was chosen to achieve a cohort target size of 200 patients who sequentially presented to the neurosurgical center. This number was chosen after discussion with a data engineer to provide sufficient data for evaluation of the NLP to be used, which had previously undergone extensive supervised and self-supervised validation on large biomedical datasets. Patients were excluded from the study if they had non-elective surgery or a non-degenerative cause of spinal disease, and if they did not have clinic letters saved to the EHR.

### Data sources and measurements

Unstructured written information from clinic letters stored within the EHR were anonymized and extracted by the information retrieval platform CogStack [13]. The CogStack platform was developed specifically to comply with the strict data governance policies of the national health service (NHS) [14]. As there may have been several clinic letters prior to the surgery, it was decided that the most detailed clinic letter was extracted for each patient prior to surgery. In addition, demographic information–age at clinic, sex and diagnosis was recorded from the EHR.

### MedCAT development

MedCAT is a ‘generalist’ medical NLP algorithm that uses machine learning (ML) to perform named entity recognition (NER). The goal of the NER is to extract information about specific types of entities (diagnoses, symptoms, or procedures) in written text that it can link to SNOMED-CT terms.

MedCAT was trained through a combination of supervised and self-supervised ML techniques, and the evaluation of concept recognition was conducted using various publicly accessible datasets, including MedMentions [15], ShARe/CLEF 2014 Task 2 [16], and MIMIC-III [17]. Additional validation was performed using electronic health records (EHR) from three major University hospitals in the UK [18]. Moreover, MedCAT underwent supervised learning in previous neurosurgical projects focusing on hydrocephalus [19] and skull base neurosurgery.

### Pipeline evaluation

**Fig. 1:**
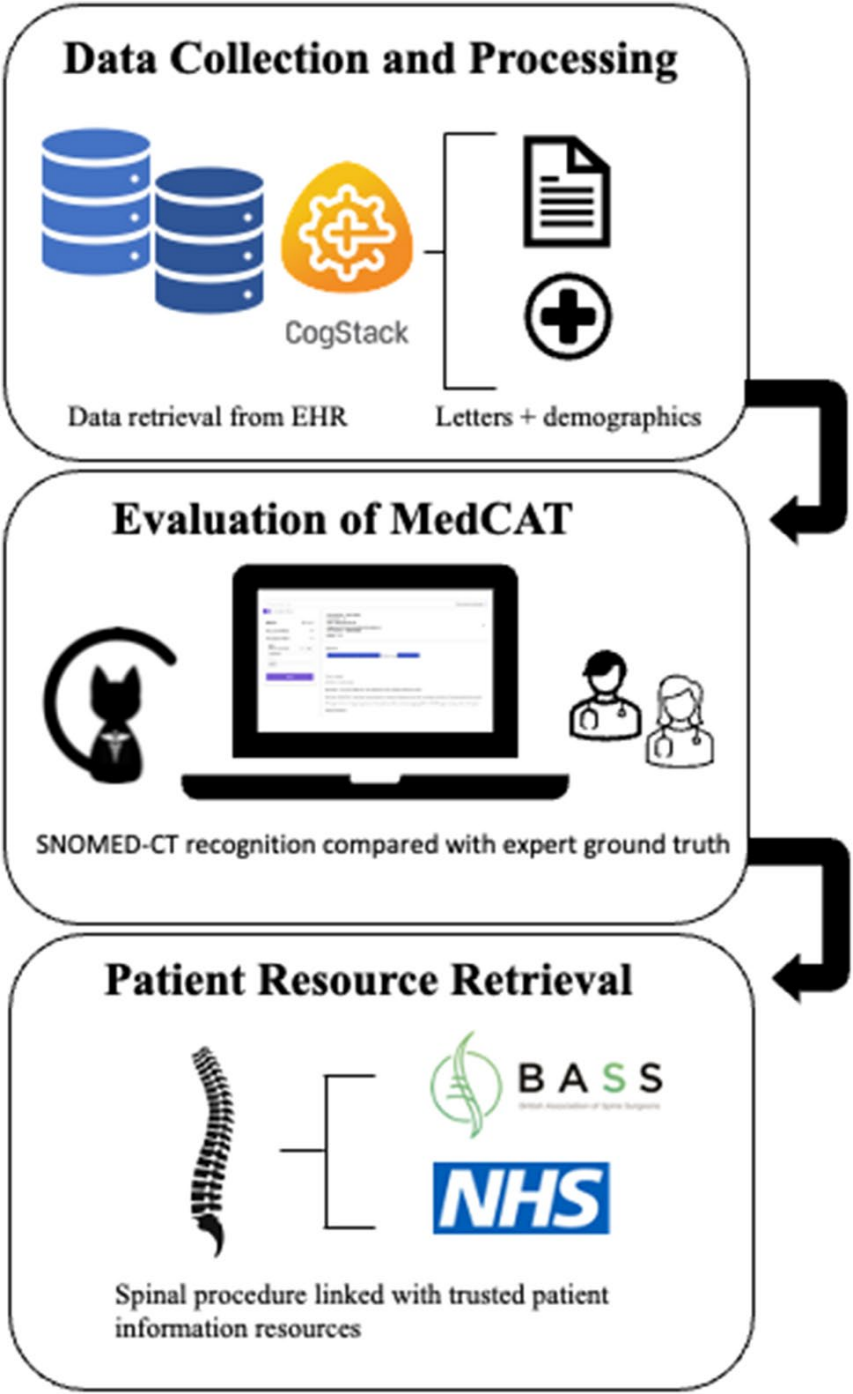
Study workflow

The MedCAT analysis was carried out in distinct stages (Fig. 1). Firstly, two neurosurgeons (JP, JB) reviewed the last three clinic letters prior to surgery for patients (N = 199) undergoing elective degenerative spine surgery and identified the most detailed of these clinic letters to label the documented spinal surgical procedure. In each clinic letter, the neurosurgeons labelled any descriptions of a surgical procedure in the letter (multi-label) and the single proposed surgical procedure that was offered to the patient. Secondly, MedCAT was tested on its ability correctly link descriptions of surgical procedures to the correct SNOMED-CT term from a list agreed upon by the neurosurgical doctors (Supplement 1). Multiple SNOMED-CT terms may be identified from each clinic letter due to clinicians repeating procedure terms or describing other procedures (e.g., discussing alternative options). This was compared against the surgical procedures identified by the neurosurgical doctors. True positive, false positive and false negative values were recorded for each SNOMED-CT concept. The NLP pipeline was optimized by adding in ‘rules’ (Supplement 2) to ensure that synonyms of procedures were identified under the relevant SNOMED CT term. Thirdly, a list of trusted educational resources was manually compiled from british association of spinal surgeons (BASS) and NHS trusts websites. The surgical procedures extracted from the NLP were then used to retrieve patient-information resources, that were linked electronically to the clinic letter on each patient’s EHR.

### Data analysis

The performance of the MedCAT model was evaluated during three separate stages: base MedCAT, MedCAT refined with added rules, and resource linkage. At each stage we calculated the precision, recall and F1 score of the model. Precision measures the proportion of true positive predictions out of all the positive predictions made by the model. In the context of MedCAT, it represents the accuracy of the model in identifying a spinal procedure. Recall measures the proportion of true positive predictions out of all the actual positive instances in the dataset. In the context of MedCAT, it represents how well the model captures all the spinal procedures mentioned in the clinic letters. The F1 score is the harmonic mean of precision and recall. It provides a balance between precision and recall, giving a single value that summarizes the model’s overall performance [20].

## Results

### Summary of data

Between January and June 2022, 199 patients who underwent surgery for degenerative spine disease had their clinic letters analyzed. The most common diagnoses were lumbar degenerative disc disease (34.2%) and cervical degenerative disc disease (20.1%). The basic demographic information of this cohort shown in Table 1.

**Table 1:** Demographic data

Characteristic	N = 199^a^
Age at clinic	60 (50, 70)
*Sex*	
Female	97 (49%)
Male	102 (51%)
*Diagnosis*	
Lumbar degenerative disc disease	68 (34.2%)
Cervical degenerative disc disease	40 (20.1%)
Lumbar disc herniation	27 (14%)
Lumbar spondylolisthesis	19 (9.5%)
Cervical spondylosis	18 (9.0%)
Cervical disc herniation	16 (8.0%)
Cervical foraminal stenosis	5 (2.5%)
Thoracic degenerative disc disease	2 (1.0%)
Thoracic disc herniation	2 (1.0%)
Diffuse Idiopathic Skeletal Hyperostosis	1 (0.5%)
Lumbar foraminal stenosis	1 (0.5%)
Osteoporotic vertebral body fracture	1 (0.5%)

^a^Median (IQR); n (%)

### Concept extraction

Initially, the MedCAT tool alone was applied to the clinical letters without additional processes in the NLP pipeline. In 187 (94%) letters the base MedCAT model identified 582 SNOMED-CT surgical procedure terms with a macro-average precision = 0.93, recall = 0.86, and F1 = 0.88 (supplement 2). In 12 letters, written text in the clinic letter did not use any text that could be linked to the prespecified list of SNOMED-CT terms or synonyms of procedures.

Using the base MedCAT model, there were many terms that were not identified as SNOMED-CT terms due to variations in written language. In some cases, the official SNOMED-CT term varied hugely from the term used to describe the procedure in clinic letters. For example, ‘ACDF’ was commonly used in clinic letters to describe the SNOMED-CT term ‘cervical arthrodesis by anterior technique’. Therefore, rules were applied to the MedCAT model to refine the pipeline and improve the uptake of SNOMED-CT terms (Supplement 2).

### Pipeline refinement

The addition of ‘rules’ to identify synonyms added a further 202 surgical procedures. The optimized MedCAT model identified 784 surgical procedures, the overall macro average performance metrics were precision = 0.98, recall = 0.86, and F1 = 0.91 (Supplement 3).

### Linking extracted terms with educational resources

**Fig. 2:**
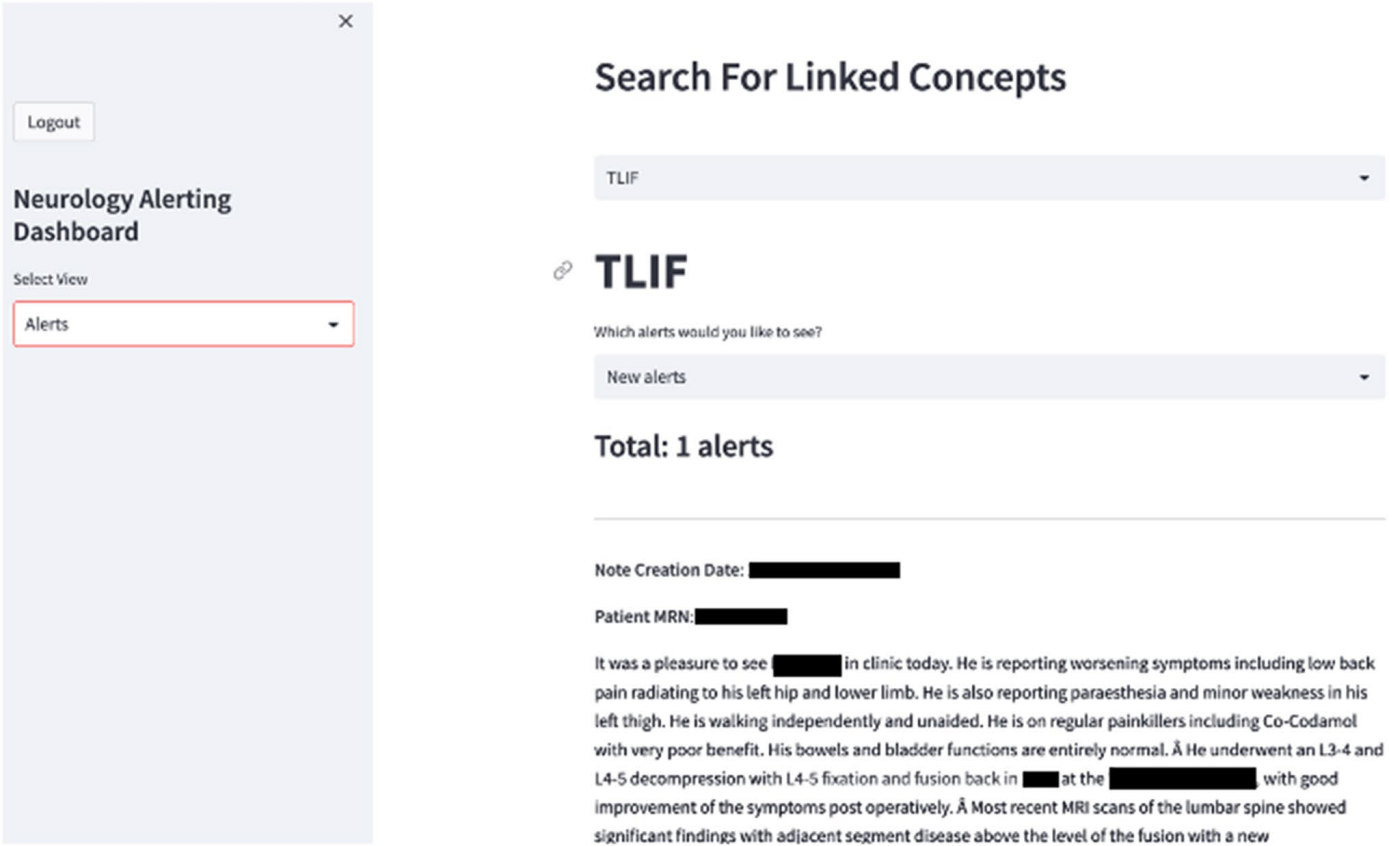
Alerting dashboard. **A** Patients with identified surgical procedures using the CogStack NLP model are highlighted to clinicians on an alerting dashboard. **B** This can then be linked with specific educational resources for patients. Personal identifiable information has been removed

**Fig. 3:**
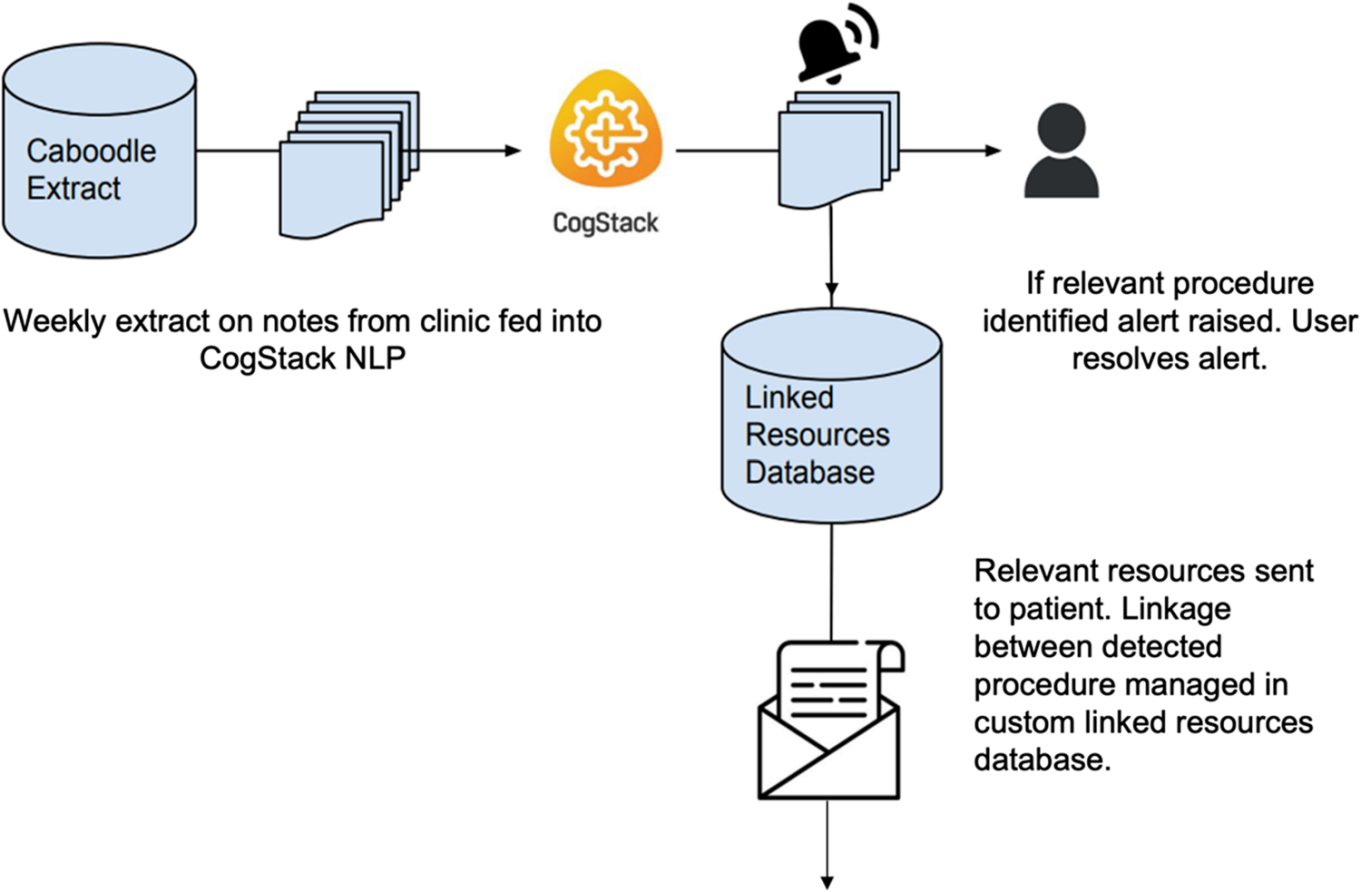
Proposed pipeline for sending educational resources to patients

A proof-of-concept alerting dashboard, integrated into the EHR, was used offline to surface patients who have been identified by MedCAT as having a spinal procedure (Fig. 2). Clinicians were able to accept or reject the alert on the dashboard, and this would in turn trigger a sequence of automated tasks that inserts/attaches relevant resources into patient’s next letter/correspondence. A proposed pipeline for sending educational resources to patients is shown in Fig. 3.

The model successfully linked extracted concepts from the letter to a specific educational resource in 157 (78%) of the 202 mutually exclusive spinal procedures performed. Overall, the macro-averages were precision = 0.99, recall = 0.87, F1 = 0.92. The precision, recall and F1 scores for this linking was calculated for each procedure (Table 2).

**Table 2:** Performance metrics for the model linking patients to educational resources

SNOMED-CT term	True positive	False positive	False negative	Precision	Recall	F1
Transforaminal interbody fusion of joint of lumbar spine	9	0	2	1.00	0.82	0.90
Excision of cervical intervertebral disc	5	0	0	1.00	1.00	1.00
Excision of lumbar intervertebral disc	1	0	0	1.00	1.00	1.00
Hemilaminectomy	7	0	0	1.00	1.00	1.00
Lumbar microdiscectomy	26	0	3	1.00	0.90	0.95
Laminotomy	1	0	0	1.00	1.00	1.00
Foraminotomy	3	0	0	1.00	1.00	1.00
Interbody fusion of lumbar spine by anterior approach	9	0	0	1.00	1.00	1.00
Primary posterior excision of cervical intervertebral disc	8	0	9	1.00	0.47	0.64
Decompression of lumbar spine	39	1	19	0.98	0.67	0.80
Osteotomy of cervical vertebra and excision of cervical intervertebral disc by anterior approach	46	1	9	0.98	0.84	0.90
Fusion of lateral lumbar interbody	3	0	1	1.00	0.75	0.86

## Discussion

### Principal findings

This study evaluated the use of an NLP model to extract SNOMED-CT terms from clinic letters. By adding simple additional processes to the pipeline, it was possible to link the identified surgical procedure with specific educational resources for patients.

Firstly, the base MedCAT model performed well without dedicated training on the dataset as indicated by precision = 0.93, recall = 0.86, and F1 = 0.88. Following refinement of the pipeline to identify synonyms of the SNOMED-CT terms, the precision of MedCAT model improved, but the recall score remained unchanged (precision = 0.98, recall = 0.86, and F1 = 0.91). This is to say that the accuracy of the MedCAT model in identifying SNOMED-CT terms improved, but the overall number of SNOMED-CT terms it identified remained unchanged. These findings collectively affirm MedCAT’s potential as a valuable tool for extracting and categorizing medical information from clinic letters, offering promising prospects for integration into clinical practice. The model's capacity to incorporate rules dynamically through a user interface, improving precision in real-time, renders it especially attractive for supporting data collection processes in healthcare settings.

Secondly, dedicated training is not required for NLP models that have been previously trained on a large volume of clinical documents, even when applied to a highly-specialist area such as spinal neurosurgery. The MedCAT model was trained with supervised and self-supervised ML on several publicly available patient databases with additional validation using EHR from three major University hospitals in the UK [18]. However, the MedCAT model did not undergo any prior training specific to spinal neurosurgery, which indicates that ‘generalist’ NLP may perform well in biomedical settings without prior training. This supports the use of a widely available Trusted Research Environments (TSE), such as CogStack, that have integrated NLP tools and can be distributed among multiple different healthcare settings, as opposed to developing multiple TSEs for each separate setting.

Thirdly, while the MedCAT model performed well at linking written text to SNOMED-CT concepts it performed less well at retrieving relevant educational resources for patients. The MedCAT model couldn’t distinguish between past spinal surgeries mentioned in clinic letters and upcoming proposed spinal procedures. Other, errors occurred when letters contained the process of discussion when considering multiple surgical options, the MedCAT model would identify each of the procedures mentioned as a separate proposed spinal procedure, despite only a single operative plan being decided upon. The result was that 78% of letters could be linked directly to a relevant educational resource. We acknowledge that clinicians currently have a higher performance to provide educational resources. However, the current practice involves clinicians manually searching the internet for resources and printing them during consultations, which poses several challenges: (1) dependence on clinicians’ memory, (2) time-consuming, (3) lacks scalability, and (4) internet resources may be variable in quality and subject to change. Further development is required of the model in interpreting letters with multiple procedural names and an overseeing clinician is recommended to ‘accept’ or ‘reject’ recommended resources, to ensure their relevance. Once functional, this clinical tool will be highly scalable to other healthcare settings, relieving clinicians of the burden of remembering and manually sourcing trusted educational resources.

Fourthly, we demonstrated that use of NLP models based on NER linking with SNOMED-CT nomenclature is limited as it does not reflect the way diagnoses are communicated to patients in a clinical setting. We found that a higher false negative rate occurred for decompression of the lumbar spine because the MedCAT NLP identified the verb ‘decompress’ as a procedural name–decompression of lumbar spine. As decompress is common language to use when describing a large variety of spinal operations, it led to a higher false negative rate. In addition, neurosurgeons used multiple different ways to describe the same spinal procedure. To account for this multiple ‘rules’ were created for each surgical procedure to link synonyms and descriptions to SNOMED-CT terms and prevent false negatives. Despite this, 12 letters used descriptive of procedures that could not be linked to SNOMED-CT terms. The heterogeneous descriptions of surgical procedures in spinal surgery represent a well-recognized problem, lacking a systematized language for effective communication. The resulting inconsistent and varied nomenclature can lead to confusion amongst patients, clinicians, and researchers [21]. A recent paper systematically analyzed the nomenclature used in the literature to describe a lateral interbody fusion procedure and identified 72 distinct ways [22]. There have been recent attempts to standardize the use of terms [23], but our study has indicated that it is an ongoing problem. We acknowledge that large language models exhibit potential advantages in interpreting text, particularly in domains with extensive language variations, such as spinal clinic letters. However, it is crucial to recognize that, at present, the integration of these large language models poses challenges in complying with the stringent data governance policies of the NHS and other healthcare systems.

### Strengths and limitations of the study

In this study, we demonstrate the value of NLP in a preoperative setting to identify surgical procedures, which can be linked with educational resources. The use case described in this study is completely novel and has wide-reaching implications for patients. The MedCAT NLP used required no dedicated training, is low cost and will be widely available to any healthcare system that uses EHR.

The data was collected from a single center, and the results may not be generalizable to other centers which clinical letters are not written in English or EHR are not available. Another limitation is that the NLP was used ‘offline’ and it is unclear if the tool will have clinical benefit for patients. The MedCAT tool now needs to be implemented into clinical practice to investigate the extent it will benefit patient’s understanding of surgical procedures.

## Conclusions

This study demonstrates the ability for an NLP algorithm, with no prior task specific training to identify surgical procedures from pre-operative clinical letters with high precision, which can be linked with specific patient education resources. Errors in the model arose due to variations in terminology used to describe spinal procedures and the model being unable to differentiate previous from future surgical procedures. Further development the NLP algorithm may lead to improved performance when linking surgical procedures with relevant educational resources. This study clinical implications for improving patient understanding of surgical procedures and empowering them engage in shared decision making.

### Supplementary Information

Below is the link to the electronic supplementary material.

Supplementary file1 (DOCX 28 KB)
