# Supplementary material for: Early evaluation of a natural language processing tool to improve access to educational resources for surgical patients
Source: Eur Spine J. 2024 May 30;33(7):2545–52. doi: 10.1007/s00586-024-08315-5 (PMC11269391; doi:10.1007/s00586-024-08315-5)
Supplement: Supplementary file 1 — Supplementary file1 (DOCX 28 KB) [file 586_2024_8315_MOESM1_ESM.docx]

# Supplement

**Supplement 1. Prespecified, relevant SNOMED-CT terms**

| **SNOMED-CT term** | **Code** |
| --- | --- |
| Cervical discectomy | 239541009 |
| Lumbar microdiscectomy | 260649000 |
| Osteotomy | 150062003 |
| Osteotomy of cervical vertebra and excision of cervical intervertebral disc by anterior approach | 439793006 |
| Transforaminal interbody fusion of joint of lumbar spine (TLIF) | 1156467004 |
| Fusion of lateral lumbar interbody (procedure) | 840279003 |
| Interbody fusion of lumbar spine by anterior approach | 719217005 |
| Decompression | 424128006 |
| Decompression of lumbar spine | 302129007 |
| Discectomy | 3418002 |
| Primary posterior excision of cervical intervertebral disc (procedure) | 178523003 |
| Excision of cervical intervertebral disc | 239541009 |
| Laminotomy | 261540001 |
| Fusion procedure | 122501008 |
| Fixation | 1431002 |
| Hemilaminectomy | 260648008 |
| Foraminotomy | 62409009 |
| Decompression operation | 410803008 |
| Excision of lamina of vertebra | 387731002 |
| Excision of lumbar intervertebral disc | 239542002 |
| Primary decompression of thoracic spine | 302127009 |
| Excision of lamina of cervical vertebra (procedure) | 445429009 |

**Supplement 2. Base MedCAT model.**

| **SNOMED-CT term** | **Count** | **True positives** | **False Positives** | **False negatives** | **Precision** | **Recall** | **F1** |
| --- | --- | --- | --- | --- | --- | --- | --- |
| Fusion procedure | 156 | 134 | 22 | 0 | 0.86 | 1.00 | 0.92 |
| Excision of cervical intervertebral disc | 48 | 48 | 0 | 7 | 1.00 | 0.87 | 0.93 |
| Excision of lamina of vertebra | 42 | 39 | 3 | 0 | 0.93 | 1.00 | 0.96 |
| Decompression | 213 | 203 | 10 | 10 | 0.95 | 0.95 | 0.95 |
| Foraminotomy | 11 | 9 | 2 | 1 | 0.82 | 0.90 | 0.86 |
| Fixation | 18 | 15 | 3 | 2 | 0.83 | 0.88 | 0.86 |
| Interbody fusion of lumbar spine by anterior approach | 6 | 6 | 0 | 0 | 1.00 | 1.00 | 1.00 |
| Decompression operation | 18 | 18 | 0 | 0 | 1.00 | 1.00 | 1.00 |
| Lumbar microdiscectomy | 36 | 34 | 2 | 15 | 0.94 | 0.69 | 0.80 |
| Transforaminal interbody fusion of joint of lumbar spine | 6 | 6 | 0 | 12 | 1.00 | 0.33 | 0.50 |
| Chondrectomy of spine | 17 | 15 | 2 | 0 | 0.88 | 1.00 | 0.94 |
| Laminotomy | 1 | 1 | 0 | 0 | 1.00 | 1.00 | 1.00 |
| Hemilaminectomy | 3 | 3 | 0 | 0 | 1.00 | 1.00 | 1.00 |
| Excision of lumbar intervertebral disc | 3 | 2 | 1 | 4 | 0.67 | 0.33 | 0.44 |
| Excision of lamina of cervical vertebra | 4 | 4 | 0 | 0 | 1.00 | 1.00 | 1.00 |

**Supplement 3. MedCAT Rules.**

| **Rule** | **MedCAT Label** |
| --- | --- |
| Anterior cervical decompression | Osteotomy of cervical vertebra and excision of cervical intervertebral disc by anterior approach |
| Anterior cervical discectomy and cage fusion | Osteotomy of cervical vertebra and excision of cervical intervertebral disc by anterior approach |
| Anterior cervical discectomy & cage fusion | Osteotomy of cervical vertebra and excision of cervical intervertebral disc by anterior approach |
| ACD | Osteotomy of cervical vertebra and excision of cervical intervertebral disc by anterior approach |
| ACDF | Osteotomy of cervical vertebra and excision of cervical intervertebral disc by anterior approach |
| Anterior cervical osteophytectomy | Osteotomy of cervical vertebra and excision of cervical intervertebral disc by anterior approach |
| Anterior decompression and fusion | Osteotomy of cervical vertebra and excision of cervical intervertebral disc by anterior approach |
| Anterior Cervical Cord Decompression and cage fusion | Osteotomy of cervical vertebra and excision of cervical intervertebral disc by anterior approach |
| C5/6 posterior decompression | Primary posterior excision of cervical intervertebral disc |
| Posterior lumbar interbody fusion | Fusion of lateral lumbar interbody (procedure) |
| Extreme lateral interbody fusion | Fusion of lateral lumbar interbody (procedure) |
| XLIF | Fusion of lateral lumbar interbody (procedure) |
| L3-L4 interbody fusion | Fusion of lateral lumbar interbody (procedure) |
| TLIF | Transforaminal interbody fusion of joint of lumbar spine (TLIF) |
| Lumbar decompression surgery | Decompression of lumbar spine |
| Lumbar decompression | Decompression of lumbar spine |
| L3-L4 decompression | Decompression of lumbar spine |
| L4-L5 decompression | Decompression of lumbar spine |
| L5-S1 decompression | Decompression of lumbar spine |
| Decompression the L2-3 and L3-4 levels | Decompression of lumbar spine |
| Lumbar spine decompression | Decompression of lumbar spine |
| Tubular minimal access decompression at the L4-5 level | Decompression of lumbar spine |
| L4-5 decompression | Decompression of lumbar spine |
| L4/5 decompression | Decompression of lumbar spine |
| L3-4 decompression | Decompression of lumbar spine |
| Decompression of L4/5 | Decompression of lumbar spine |
| L3-4 surgical decompression | Decompression of lumbar spine |
| Decompression of the right sided L5 nerve root | Decompression of lumbar spine |
| Decompression, at the L2/3 | Decompression of lumbar spine |
| Lumbar microsurgical decompression | Lumbar microdiscectomy |
| Microsurgical decompression | Lumbar microdiscectomy |

**Supplement 3. Optimised MedCAT model.**

Shows the additional surgical procedures that were identified through synonym ‘rules’.

| **SNOMED-CT term** | **True positive** | **False positive** | **False negative** | **Precision** | **Recall** | **F1** |
| --- | --- | --- | --- | --- | --- | --- |
| Transforaminal interbody fusion of joint of lumbar spine | 8 | 0 | 0 | 1 | 1 | 1 |
| Primary posterior excision of cervical intervertebral disc | 2 | 0 | 5 | 1 | 0.29 | 0.44 |
| Decompression of lumbar spine | 43 | 6 | 17 | 0.88 | 0.72 | 0.79 |
| Osteotomy of cervical vertebra and excision of cervical intervertebral disc by anterior approach | 77 | 0 | 7 | 1 | 0.92 | 0.96 |
| Fusion of lateral lumbar interbody | 18 | 0 | 3 | 1 | 0.86 | 0.92 |
